# Mechanisms of Mining-Induced Surface Hazards Beneath Steep Ridge-Type Mountain Geometry

**DOI:** 10.3390/s26041260

**Published:** 2026-02-14

**Authors:** Guangyao Song, Xin Yao, Xuwen Tian, Zhenkai Zhou, Xiaoqiang Chen

**Affiliations:** 1 Institute of Geomechanics, Chinese Academy of Geological Sciences, Beijing 100081, China; songguangyao24@mails.ucas.ac.cn (G.S.); 20201200455@csuft.edu.cn (X.T.); zhouzhenkai@mail.cgs.gov.cn (Z.Z.); cxq@email.cugb.edu.cn (X.C.); 2 Key Laboratory of Active Tectonics and Geological Safety, Ministry of Natural Resources, Beijing 100081, China

**Keywords:** coal mining, surface hazards, SBAS-InSAR, subsidence, GNSS

## Abstract

**Highlights:**

**What are the main findings?**

- Coal mining beneath steep ridge-type mountain geometry can induce significant surface deformation even at great depths due to topographic and lithological amplification effects.
- Mining-induced surface deformation in mountainous areas does not necessarily trigger large-scale slope failure and may gradually stabilize through natural adjustment processes.

**What are the implications of the main findings?**

- Integrated SBAS-InSAR, GNSS, UAV, and field investigations provide a reliable framework for detecting and interpreting mining-induced deformation in steep ridge-type mountain geometry in mountainous terrain.
- The strong coupling between steep ridge-type mountain geometry and multi-face coal extraction reveals a compound hazard chain, offering guidance for disaster prevention and risk management in similar mountainous mining regions.

**Abstract:**

Coal mining in plain regions and its related surface subsidence and geological hazards have been extensively studied, whereas research on mining-induced hazards in mountainous areas remains limited. This knowledge gap has contributed to the frequent occurrence of mining disasters, particularly under steep ridge-type mountain geometry, where deformation characteristics, large-scale slope failure risks, and mining-induced hazard mechanisms remain poorly understood. In this study, a mining area in Zhenxiong, Zhaotong, Yunnan Province, China, is investigated using SBAS-InSAR, GNSS observations, UAV surveys, optical satellite imagery, and detailed field investigations. Surface hazards triggered by coal extraction are identified, and the response relationship between surface subsidence and mining activities is analyzed to reveal the development mechanisms of surface deformation beneath steep ridge-type mountain geometry. The results show that: (1) deep coal mining can still induce significant surface deformation due to the combined amplification effects of steep slopes and lithological conditions; (2) mining-induced deformation does not necessarily evolve into large-scale slope collapse and may gradually stabilize through natural adjustment processes; (3) SBAS-InSAR, validated by GNSS and field observations, provides an effective approach for detecting mining-related subsidence; (4) surface deformation in the study area is jointly influenced by multiple working faces; and (5) strong coupling between the unique steep ridge-type mountain geometry and underlying coal extraction leads to a compound disaster chain under multi-source interactions. These findings offer a critical scientific understanding of mining-induced deformation beneath steep ridge-type mountain geometry and provide important guidance for geological hazard prevention and control in similar mountainous mining areas.

## 1. Introduction

Coal resources constitute one of the most important components of the global energy sector and play an irreplaceable role in human production and daily life. China ranks first worldwide in coal production, accounting for approximately 51.7% of the global total [1], and the coal industry in Southwest China plays a particularly important role within the national coal sector [2].

Coal-bearing strata are widely distributed in Southwest China [3], where the karst mountainous region belongs to the Yangtze Platform. Owing to long-term tectonic activity and fluvial erosion, the region is characterized by highly complex and diverse topography and geomorphology, including steep high mountains, deep valleys, V-shaped gorges, and large-scale cliffs, forming extremely steep terrain conditions [4]. In addition, modern coal mining techniques are characterized by large mining heights, wide working faces, rapid advance rates, and high productivity. However, underground coal mining exerts significant negative impacts on the geological environment in mountainous areas and can trigger a series of surface geological hazards, such as collapses, landslides, debris flows, ground fissures, and surface subsidence [5,6,7]. Against this background, geological disasters occur frequently in Southwest China, particularly in extremely steep mountainous regions [8]. Typical examples include the Pusa landslide in Nayong County, Bijie City, Guizhou Province, in 2017 [9]; the Madaling landslide in Jiangzhou Town, Duyun City, Guizhou Province, in 2006 [10]; the Jiweishan landslide in Wulong, Chongqing, in 2009 [11]; the landslide in Liangshui Village, Zhenxiong County, Zhaotong City, Guizhou Province, in 2024 [12]; and the Jinping landslide in Junlian County, Yibin City, Sichuan Province, in 2020 [13].

At present, research on coal mining and its associated surface subsidence and geological hazards in plain areas is relatively established. For example, Zhang et al. (2015) [14] used DS-InSAR techniques to monitor mining-induced ground subsidence in Huainan City, China. Guéguen et al. (2009) [15] employed D-InSAR and Persistent Scatterer Interferometry (PSI) to detect surface subsidence caused by coal mining in the Northern Basin of France. Wu et al. (2025) [16] applied wide-area Interferometric Synthetic Aperture Radar techniques combined with machine learning algorithms to detect coal mining-induced surface deformation in Shanxi Province, China. However, studies on coal mining in mountainous regions with complex topographic conditions remain insufficient, particularly with respect to the mechanistic influence of mountain morphology on mining-induced surface hazards. In previous studies, a few recent works have demonstrated the feasibility of using InSAR to identify mining-related deformation in karst regions [17,18,19] and mountainous environments [20,21] of Southwest China; however, the coupling relationship between terrain geometry and the evolution of mining-induced hazards has not yet been investigated in depth. Underground mining leads to deformation, damage, and stress redistribution within the overburden, resulting in fracturing, movement, and re-equilibration of rock mass structures above the goaf [22,23]. According to classical mining subsidence theory, mining-induced surface subsidence can generally be divided, from bottom to top, into the collapse zone, fracture zone, and bending subsidence zone [24,25]. Nevertheless, many existing studies implicitly assume flat surface conditions in mining areas, thereby neglecting the differential effects introduced by complex terrain [26]. Compared with plain regions, surface subsidence in mountainous areas exhibits pronounced spatial and temporal heterogeneity and asymmetry [27], which substantially increases the complexity of slope deformation processes.

Monitoring technologies such as the Global Positioning System (GPS) and total stations have been widely applied in mining areas for surface hazard monitoring and deformation analysis. However, compared with Interferometric Synthetic Aperture Radar (InSAR), traditional monitoring techniques are constrained by high labor intensity, high cost, and limited efficiency. With continued methodological advances, InSAR has been increasingly applied to ground-subsidence and slope hazard monitoring in mining regions. Owing to its high precision, wide spatial coverage, low cost, strong temporal continuity, capability for historical deformation retrieval, and insensitivity to weather conditions, InSAR is regarded as one of the most promising techniques for ground deformation monitoring. For example, Carnec et al. (1996) [28] first applied D-InSAR to monitor surface subsidence in Saint-Étienne-de-Tinée and the Gardanne mining area, demonstrating the feasibility of InSAR for subsidence studies. Pawluszek-Filipiak et al. (2020) used D-InSAR and SBAS-InSAR to measure mining-related deformation in the Rydułtowy coal mine in Poland and systematically compared the advantages and limitations of the two methods [29]. Zhang et al. (2024) generated a nationwide surface deformation dataset for China covering 2018–2022 using 47,000 Sentinel-1 images and the SBAS-InSAR technique [30]. However, in parts of the mountainous regions of Southwest China, dense vegetation cover leads to low interferometric coherence, making it difficult to extract stable deformation signals, while frequent cloud cover and complex atmospheric conditions introduce atmospheric delays that adversely affect deformation measurements. Therefore, InSAR-based surface deformation monitoring should be integrated with GNSS observations [31] to improve reliability and enable dynamic risk monitoring in mining areas [32,33,34].

## 2. Study Area

**Figure 1:**
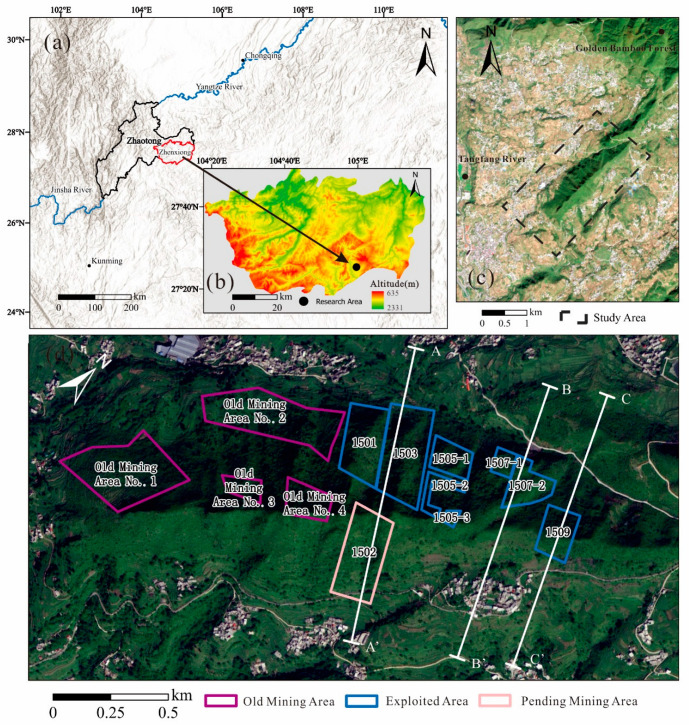
Geographical location of the study area: (**a**) location of Zhaotong City and Zhenxiong County; (**b**) digital surface model (DSM) of Zhenxiong; (**c**) satellite image of the study area; and (**d**) distribution of working faces and locations of geological cross-sections.

**Figure 2:**
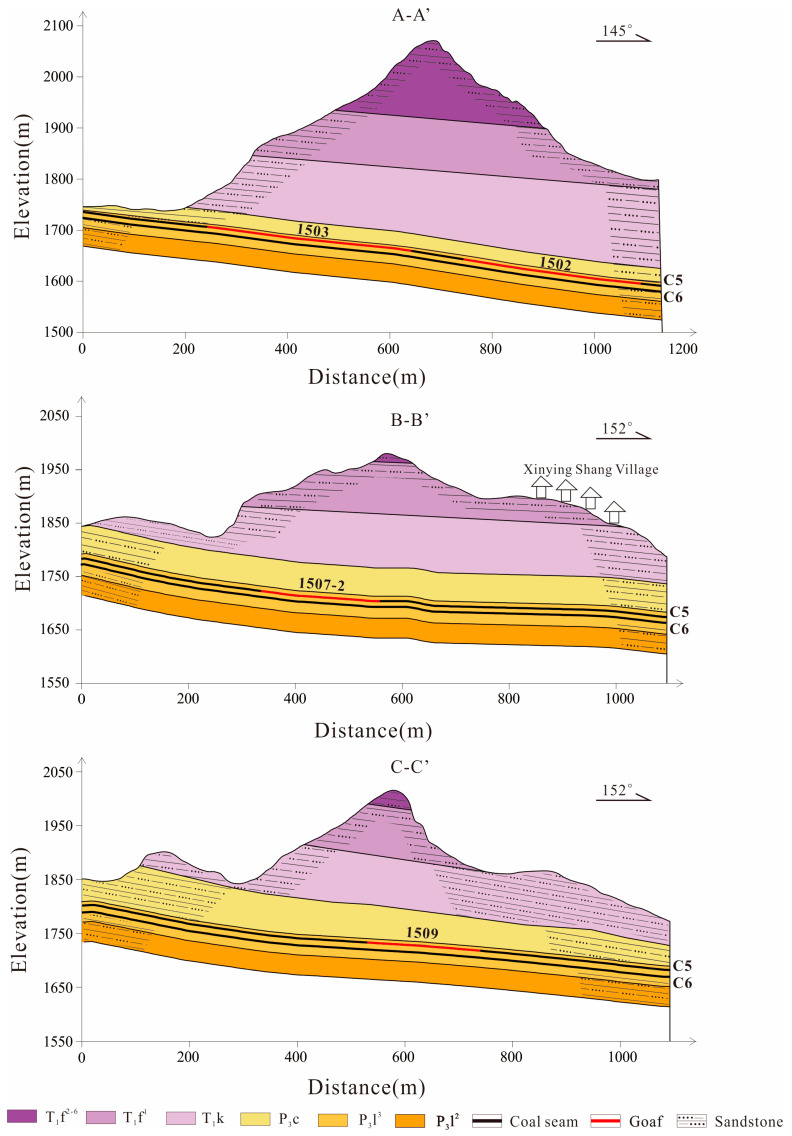
Engineering geological profile (the location is shown at line segments A–A′, B–B′, and C–C′ in Figure 1d).

The study area is located in Zhenxiong County, Yunnan Province, in the northern part of the Wumeng Mountains area. The terrain is strongly dissected, characterized by high mountains and deep valleys, with steep surface slopes generally ranging from 15° to 25° (Figure 1a,b). The western part of the study area is characterized by steep terrain, with slope angles generally ranging from 30° to 50°, and locally approaching near-vertical conditions. Based on the plan view and cross-sectional profiles of the study area (Figure 1c and Figure 2), the mountain morphology exhibits a typical steep ridge-type mountain geometry. According to geomorphological classification, the study area is characterized by a structural denudation–erosional landform. The mountain range generally trends in a northeast–southwest (NE–SW) direction, and the extension of the mountain ridges is basically consistent with the stratigraphic strike and the main tectonic structural lines. Overall, the topography is higher in the northeast and lower in the southwest. The highest elevation in the mining area and its surroundings is located at the summit of Golden Bamboo Forest Mountain in the northeastern part of the mining area, reaching an altitude of 2324.9 m. The lowest point is located at the Tangfang River in the southwestern part of the mining area, with an elevation of 1390 m, resulting in a relative relief of 934.9 m (Figure 1c). This river represents the lowest regional erosion base level, and the area is classified as a mid-mountain landform.

The mineable coal seams in the study area include the C5 and C6 seams, with average thicknesses of 1.7 m and 0.77 m, respectively (Figure 2). The coal seams have a gentle dip angle of approximately 6°. At present, only the C5 seam is being exploited, whereas the C6 seam has not been mined. Coal mining in the study area has a long history; prior to 1991, coal extraction was carried out sporadically by local villagers. According to the distribution of the working faces (Figure 1d), the No. 1 old goaf was mined from January to December 2005. From February 2011 to December 2013, the No. 2, No. 3, and No. 4 old goafs were successively mined. The mining areas from August 2021 to January 2024 are indicated by blue boundaries. Specifically, the 1501 working face was mined from August to November 2021; the 1503 working face from December 2021 to May 2022; the 1505-1, 1505-2, and 1505-3 working faces from June 2022 to July 2023; and the 1507-1 and 1507-2 working faces from August 2022 to August 2023. The planned areas to be mined are delineated by pink boundaries. The mining schedule of the 1502 working face has not yet been determined.

## 3. Materials and Methods

**Figure 3:**
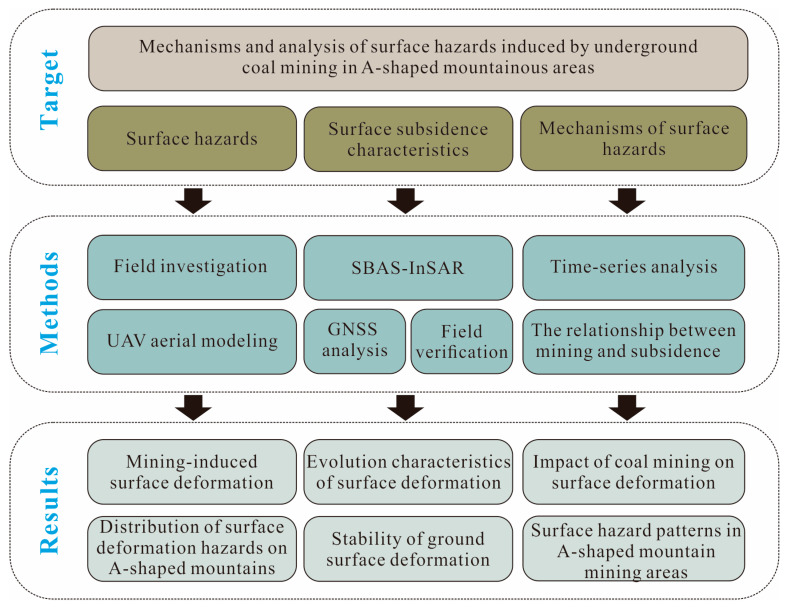
The technical approach in this study.

Figure 3 illustrates the technical workflow of this study. Field investigations, UAV aerial photogrammetric modeling, and InSAR time-series processing were employed to investigate ground surface hazards. Within this research framework, the study systematically explores the impacts of coal mining on surface deformation in steep ridge-type mountain geometry areas, as well as the associated hazard development patterns.

### 3.1. UAV and Field Investigations

**Figure 4:**
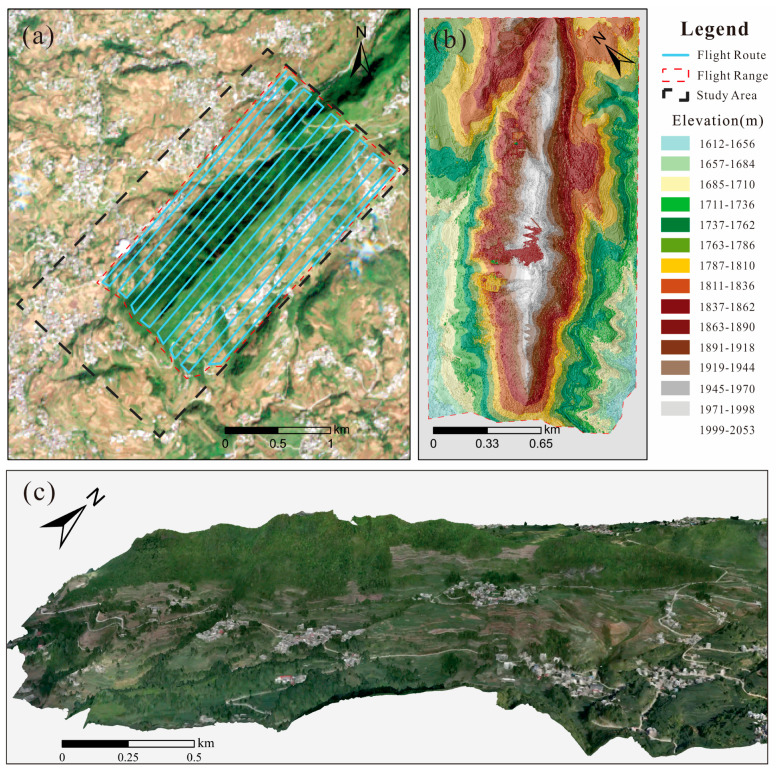
Results of the UAV photogrammetric survey: (**a**) UAV flight route planning; (**b**) DSM generated from the UAV survey; and (**c**) three-dimensional realistic model generated from UAV images.

From May to August 2024, unmanned aerial vehicle (UAV) photogrammetric surveys and detailed field investigations were conducted in the study area. The UAV platform used in this study was the DJI MATRICE 300 RTK, manufactured by DJI ((Da-Jiang Innovations Science and Technology Co., Ltd.), Shenzhen, N/A, China), which is equipped with high-precision surveying and mapping capabilities. During the UAV survey, the flight parameters were set as follows: a flight altitude of 400 m, a flight speed of 8 m/s, a lateral overlap of 70% (Figure 4a), and a longitudinal overlap of 80%. A total of 1800 aerial images were acquired and subsequently processed using DJI Terra V4.0.1 to generate a digital surface model (DSM) (Figure 4b) and a three-dimensional visualization model with a spatial resolution of 0.05 m (Figure 4c).

**Figure 5:**
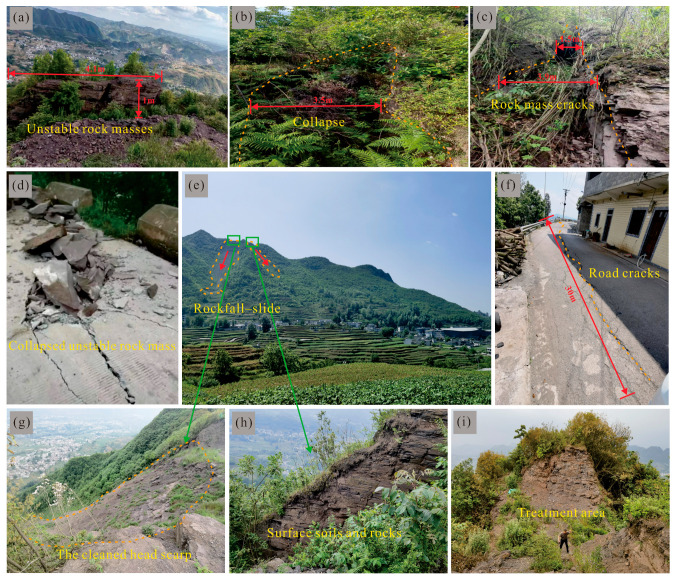
Field investigation photographs of geological hazards in the study area: (**a**,**d**) unstable rock masses related to rockfall hazards and the damage to the roadway caused by falling rocks; (**b**) collapse; (**c**,**f**) rock mass cracks and ground cracks, as well as roadway damage induced by fissures; (**e**,**g**,**h**) two rockfall–slide sites and photographs of the rockfall–slide cleaned head scarp and the surface soil and rock mass of the head scarp; (**i**) area where unstable rock masses have been cleared.

Based on multiple detailed field investigations and a careful analysis of the study area’s distinctive steep ridge-type mountain geometry, the predominant hazards in the study area are rockfall–slide, collapse, and ground fissures. Cracks in the mountain mass are mainly distributed linearly along the ridge, and two large fissures are located within the planned 1502 working face (Figure 4c and Figure 5c). In addition, three rockfall-prone zones were identified, and falling rock from two of these hazardous zones has damaged the roadway (Figure 5f). The collapsed/failed unstable rock masses have since been cleared or are currently under monitoring (Figure 4c and Figure 5a,d,e,g–i). Two ground-subsidence sites were also identified; both have not yet affected roads or residents (Figure 4c and Figure 5b). Nevertheless, fissures on the ridge and slope, rockfalls, and ground subsidence have the potential to damage buildings and roads and pose a threat to local residents’ safety.

### 3.2. InSAR Time-Series Analysis

Interferometric Synthetic Aperture Radar (InSAR) is a high-precision microwave remote sensing technique for detecting subtle ground surface deformation by generating interferometric phase information from two coherent Synthetic Aperture Radar (SAR) images acquired over the same area. The Small Baseline Subset InSAR (SBAS-InSAR) technique is a classical time-series InSAR approach [35], which optimizes SAR image combinations using a multi-master strategy based on short temporal and spatial baselines. This method effectively reduces atmospheric delay errors and suppresses topographic phase effects, thereby enabling the detection of small-amplitude surface deformation [36].

In this study, the SBAS-InSAR technique was applied to retrieve surface deformation. The topographic phase was removed using the Shuttle Radar Topography Mission digital elevation model (SRTM DEM) with a spatial resolution of 30 m and a vertical accuracy of ±10 m, obtained from USGS EarthExplorer. The parameters of the SAR imagery used are shown in Table 1. The SAR dataset consists of 84 ascending-orbit scenes acquired by Sentinel-1 between 5 January 2022 and 1 January 2025. The range and azimuth multi-looking factors were set to 2 × 5, respectively. The temporal baseline was constrained to the revisit interval of three consecutive SAR acquisitions, and the average perpendicular baseline was 65.222 m. It should be noted that all InSAR-derived deformation results in this study are presented along the radar line of sight (LOS).

### 3.3. Key Position GNSS Analysis

**Figure 6:**
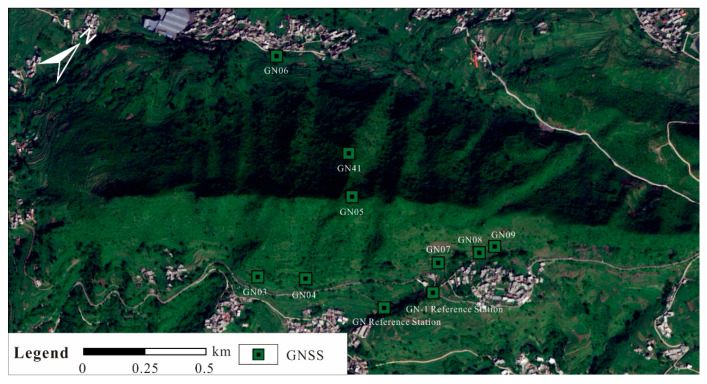
GNSS monitoring point distribution.

In geological hazard monitoring, the GNSS technique has become an indispensable monitoring approach due to its advantages of high precision, all-weather capability, and automation. GNSS sensors are deployed on the ground to measure the deformation of characteristic points, and they are particularly suitable for the real-time automatic monitoring of landslides with high risk or intense deformation (i.e., high deformation rates or accelerating deformation), enabling timely analysis and early warning during short-term imminent and emergency periods [37]. However, most surface deformation monitoring techniques are susceptible to disturbances from atmospheric conditions, complex topography, vegetation cover, and human activities. Therefore, the selection of monitoring site locations is directly related to the accuracy and reliability of the monitoring data [38]. In the study area, a total of 10 GNSS instruments were installed (Figure 6), and monitoring data collected from 5 June 2024 to 31 January 2025 were used for subsequent stability analysis.

## 4. Results

### 4.1. Mining-Induced Surface Deformation

**Figure 7:**
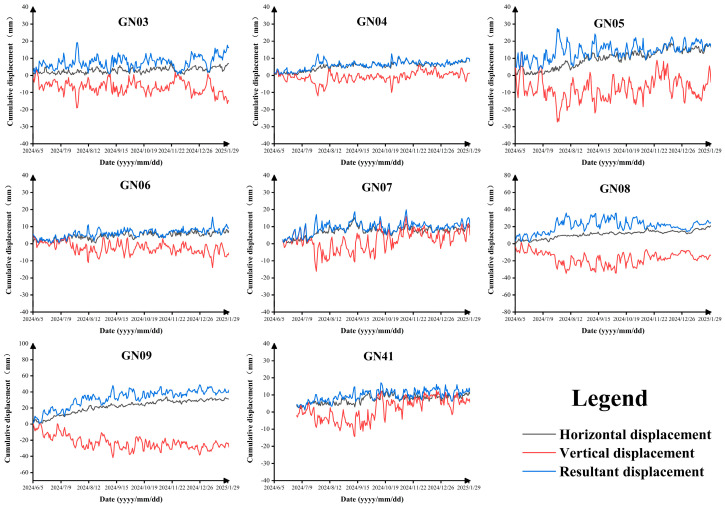
The results of surface deformation monitoring using instruments in coal mines.

GNSS monitoring data from eight sites in the study area were collected for the period from 5 June 2024 to 29 January 2025 (Figure 6). The overall cumulative deformation is small, generally ranging from 10 to 30 mm, and has shown a clear converging trend over the past six months (Figure 7).

At the GN03, GN04, and GN05 stations, which monitor slope deformation on the eastern side of Longwang Mountain, the cumulative displacements are 11.3 mm, 9.5 mm, and 21.7 mm, respectively. These values generally fall within the range of the natural creep of steep slopes, and only the summit station GN05 represents residual mining-induced rock mass movement. At the GN07, GN08, and GN09 stations, which also monitor slope deformation on the eastern side of Longwang Mountain, the cumulative displacements are 6.3 mm, 21.3 mm, and 44.2 mm, respectively. These magnitudes are characteristic of slow, natural deformation of loose accumulation slopes and do not indicate precursors of major hazardous landslides. At the GN06 and GN41 stations, which monitor slope deformation on the western side of Longwang Mountain, the cumulative displacements are only 6.9 mm and 7.7 mm, respectively, also corresponding to the natural creep of steep slopes. In summary, the GNSS monitoring results indicate that deformation at all sites on Longwang Mountain is generally minor and exhibits a converging trend. Except for the normal creep of landslides under natural conditions, the other slopes have, overall, returned to a state of natural deformation.

### 4.2. The Evolution of Surface Deformation

**Figure 8:**
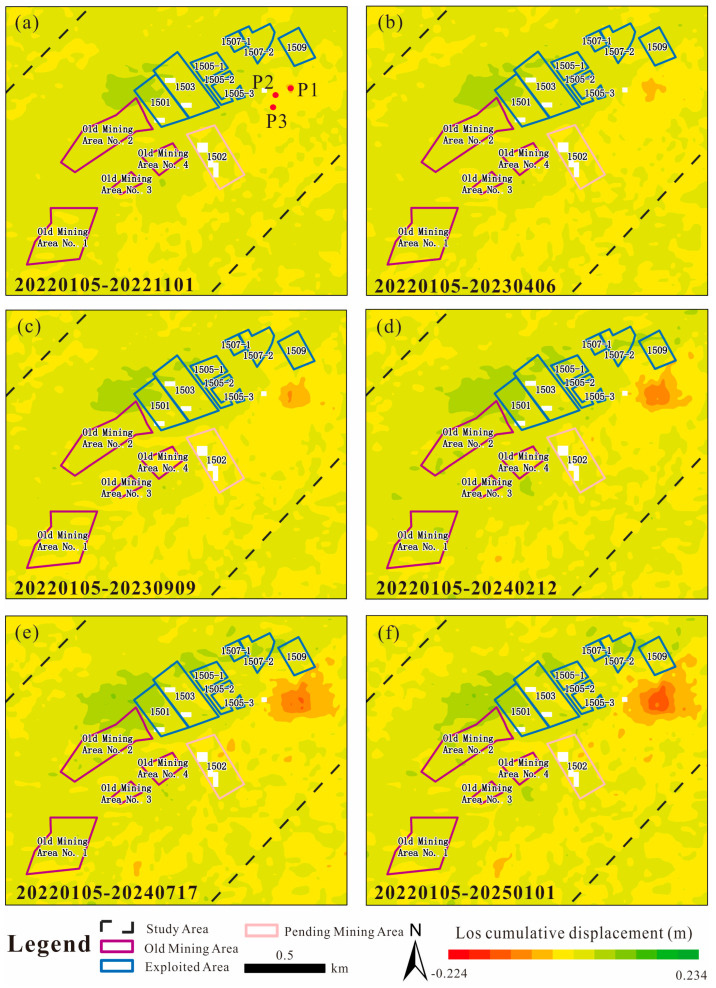
Evolution of cumulative deformation in the key area of the study site from January 2022 to January 2025; the locations of monitoring points for the time-series deformation curves are shown in (**a**).

Overall, based on nearly three years of Sentinel-1 InSAR deformation observations from 5 January 2022 to 1 January 2025, surface deformation in the mining area is mainly concentrated in a single zone. The subsidence area exhibited minor deformation signals between 5 January and 1 November 2022 (Figure 8a). From 5 January 2022 to 6 April 2023 (Figure 8b), the deformation gradually expanded, and during the subsequent period, the deformation rate showed an accelerated trend (Figure 8c–f).

The mountain in the study area is characterized by a distinctive steep ridge-type mountain geometry, with rock strata dipping toward 101–105° and dip angles ranging from 5° to 12°. From August 2021 to January 2024, six working panels were successively excavated in the study area. By jointly analyzing the SBAS-InSAR deformation results and the excavation schedule of each working panel, it is demonstrated that coal mining activities constitute the primary controlling factor responsible for surface subsidence deformation. Across the entire mining area, only one prominent large-scale deformation zone has developed, while no obvious regional deformation is observed elsewhere. This further indicates that the current mining-induced ground movement in the study area is spatially limited and the deformation pattern is relatively simple.

**Figure 9:**
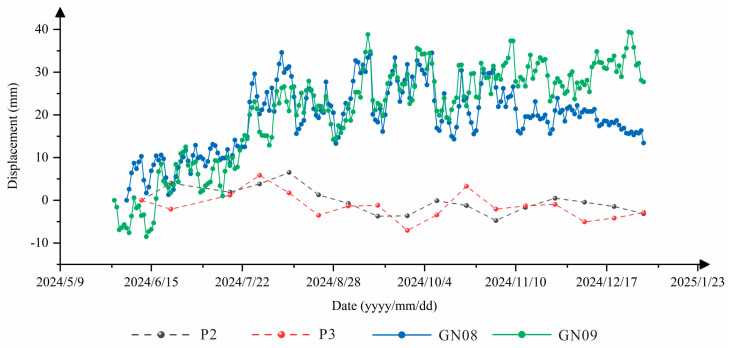
Comparison of the SBAS-InSAR results and the GNSS data.

To validate the accuracy of the SBAS-InSAR measurements, GNSS monitoring data acquired between 31 May 2024 and 1 January 2025 were compared with SBAS-InSAR results obtained between 11 June 2024 and 1 January 2025. The deformation at the initial date was set to zero. In theory, the positions of the GNSS observation points GN08 and GN09 (Figure 6) should coincide exactly with those of the SBAS-InSAR measurement points P2 and P3 (Figure 8a). The GNSS data were collected at a temporal interval of 1 day, whereas the SBAS-InSAR monitoring results have a temporal resolution of 12 days. The comparison results are shown in Figure 9.

As shown in Figure 9, the cumulative displacements at the corresponding points exhibit a high degree of consistency in their overall trends, although they do not completely coincide. In addition, the GNSS observations show certain differences in variability compared with the SBAS-InSAR measurements. This discrepancy is mainly attributed to the inconsistency in temporal resolution between the two measurement techniques, as well as the fact that SBAS-InSAR measures deformation along the radar line-of-sight direction, whereas the GNSS deformation measurements use the resultant displacement component. Despite these limitations and the resulting deviations between the two datasets, the SBAS-InSAR deformation measurements in this area still demonstrate relatively high accuracy and reliability. Therefore, the results of this study provide important reference values for deformation monitoring in steep ridge-type mountain geometry and can offer a theoretical basis for similar studies.

### 4.3. Mining-Induced Surface Hazard Inventory

**Figure 10:**
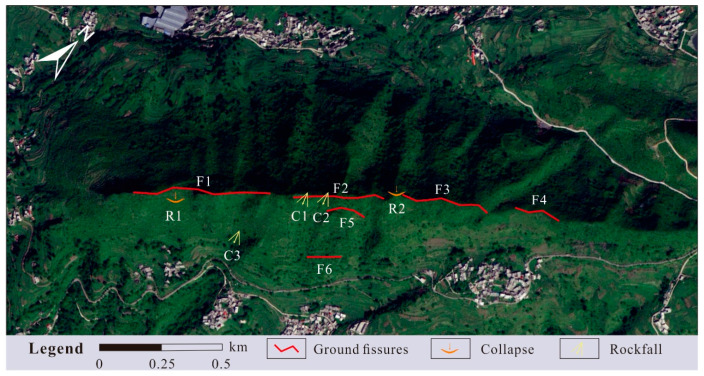
Distribution map of geological hazards based on optical satellite imagery.

Surface deformation in the study area is mainly characterized by the development of multiple tension cracks at the mountaintop (Figure 5c), locally distributed ground collapse pits (Figure 5b), and unstable rock masses occurring along steep cliff sections (Figure 5a). A total of 11 deformation features have been identified on the steep ridge-type mountain geometry in the study area, including six ground fissures, three potential rockfalls, and two ground collapse zones (Figure 10).

Among the ground fissures developed on the slope, those located in the middle to upper part of the slope generally exhibit extension lengths of approximately 40–60 m, with widths ranging from 0.8 to 2.0 m and visible depths of about 0.1–2.0 m. Some fissures exhibit a vertical displacement (downthrow) of approximately 1.0 m along segments parallel to the slope strike. The orientations of the fissures are irregular: the lower segments locally extend in a near-vertical direction, whereas the upper segments bend and become approximately parallel to the mountain trend. In addition, a discontinuously developed fissure exceeding 300 m in length is observed near the mountaintop, with widths ranging from 0.5 to 2.5 m and visible depths generally between 0.1 and 1.0 m, reaching a maximum depth of 3.0 m. This fissure strikes consistently with the mountain trend and extends in a northeast–southwest (NE–SW) direction.

Among the three identified potential rockfalls, the C2 exhibits the highest hazard level, which is located on the southeastern side of the mountain. Due to intense weathering of the steep cliff face, the development of unloading fractures and weathering-induced joints, and the poor integrity of the rock mass, its stability is significantly reduced, indicating a high potential for collapse. Based on deformation characteristics, the unstable rock mass exhibits an approximate width of 50 m, height of 15 m, and thickness of 5 m, with an estimated volume of about 3750 m^3^. The predicted collapse direction is 165°, and it is classified as a small-scale rockfall-prone body. If failure occurs, the collapsing rock mass would pose a direct threat to the residential area located downslope, indicating a high level of hazard and risk.

## 5. Discussion

### 5.1. On the Duration of Post-Mining Mountain Deformation and the Development of Fracture Connectivity

Underground coal mining induces the formation and collapse of goafs, which significantly affects the stability of mountain slopes and may further trigger secondary geological hazards such as landslides, rockfalls, and debris flows. The mountain in the study area exhibits a distinctive steep ridge-type mountain geometry, with bedding attitudes characterized by dip directions of 101–105° and dip angles of 5–12°. From August 2021 to January 2024, five working faces were successively excavated in the study area. By comparing the SBAS-InSAR deformation maps with the excavation schedule of each working face, it is evident that coal mining activities are the primary controlling factor responsible for the surface subsidence deformation at point P1.

During the period from August 2021 to May 2022, the 1501 and 1503 working faces were mined. Because the strata dip direction (101–105°) is oriented toward point P1, the formation of goafs due to coal extraction led to the collapse of overlying strata. Under gravitational loading, a compressive stress concentration zone developed at the slope toe downslope of point P1, and the progressive extrusion at the slope toe induced subsidence deformation in the upper part of the slope.

Subsequently, mining of the 1505-1, 1505-2, and 1505-3 working faces from June 2022 to July 2023, together with the extraction of the 1507-1 and 1507-2 working faces from August 2022 to August 2023, caused continuous deformation at point P1. When the 1509 working face was excavated between September 2023 and January 2024, the degree of deformation at point P1 further intensified.

After the cessation of mining activities, surface deformation was found to persist, indicating that in steep ridge-type mountain geometry, the deformation at the slope toe induced by underground goaf collapse has a long-lasting response time and prolonged duration. Therefore, in future coal mining activities under similar mountain conditions, continuous post-mining monitoring using appropriate deformation monitoring instruments should be implemented for an extended period after mining operations have ceased.

It is also noteworthy that no significant surface deformation was detected directly above the goaf on the InSAR deformation maps, suggesting that fractures generated by the collapse of the underground goaf did not propagate continuously through the overburden to reach the ground surface.

### 5.2. Effectiveness and Limitations of InSAR Monitoring of Coal Mining-Induced Surface and Mountain Deformation

**Figure 11:**
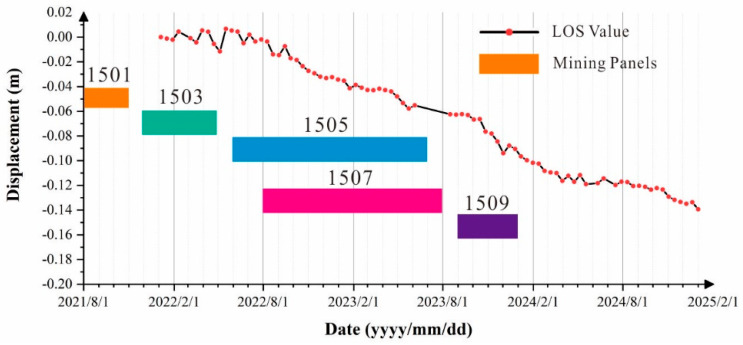
Time-series deformation curve at point P1 and the mining schedule of the working faces.

Coal mining activities can substantially disturb the ground surface and induce surface deformation. By comparing the deformation time-series curve at the maximum deformation point (P1) with the excavation schedule of the working faces (Figure 8a and Figure 11), it is observed that during active mining, the slope of the subsidence curve at P1 increases significantly, accompanied by a significant increase in cumulative settlement along the LOS direction. This change is particularly pronounced during the simultaneous mining of the 1505 and 1507 working faces and during the excavation of the 1509 working face, indicating that coal seam extraction exerts a strong influence on mountain deformation. Although point P1 is not located directly above the working faces, it is likely situated within the influence zone of the goafs formed after coal extraction. The superposition of deformation effects from multiple adjacent goafs is therefore considered to be the primary cause of the pronounced deformation observed at point P1.

However, under complex mountainous terrain conditions, the limitations of InSAR imposed by the radar line-of-sight (LOS) geometry become more pronounced, constraining both its capability to detect surface deformation and the reliability of deformation interpretation. First, InSAR can only retrieve the one-dimensional projection of surface displacement along the LOS direction, whereas actual mountain deformation typically involves multiple components, including vertical subsidence, horizontal extension, and slope-parallel movement. In steep mountainous areas, deformation directions are often controlled by slope orientation and ridge trends, with significant slope-parallel and lateral components. When these components form a large angle with the radar LOS, the sensitivity of InSAR is substantially reduced, which may lead to underestimation or even omission of deformation signals.

Second, InSAR is extremely insensitive to north–south displacement components. In practical applications, deformation is usually estimated using ascending and descending orbit data to derive vertical and east–west components, while implicitly assuming that the north–south component is negligible. In complex mountainous environments, however, this assumption does not always hold and may introduce systematic errors.

Finally, complex topography amplifies the propagation of LOS geometric errors. Steep slopes, deep valleys, and sharp ridges cause significant local variations in the radar incidence angle, resulting in the same true deformation being expressed as different LOS-projected magnitudes across neighboring pixels. Moreover, geometric distortions such as layover, shadowing, and low coherence are widespread in mountainous regions, further weakening the capability of InSAR to detect slope-parallel and lateral deformation.

Therefore, relying solely on LOS-based InSAR deformation results in complex mountainous settings entails inherent limitations. It is necessary to introduce multi-source observations, such as GNSS, for constraint and supplementation. Through integrated InSAR–GNSS analysis, directional component biases can be quantitatively evaluated, thereby improving the reliability of deformation interpretation and hazard assessment for mining-induced surface deformation in steep ridge-type mountain geometry.

### 5.3. Surface Hazard Patterns in Steep Ridge-Type Mountain Geometry Mining Areas

The steep ridge-type mountain geometry is a relatively rare geomorphic type among coal mining mountain terrains in Zhenxiong County, and, to date, there has been a lack of systematic studies on geological hazards induced by mining activities under this specific mountain morphology.

Because the active coal mining panels are located beneath the extremely steep ridge-type mountain geometry formed by Longwang Mountain and Moon Mountain, the area has become a high-risk zone for major geological hazards. In the ridge area of the study site, the exposed strata are mainly composed of siltstone interbedded with mudstone and shale of the Lower Triassic Feixianguan Formation. Within these strata, joints and unloading fractures are strongly developed, and two groups of dominant structural joints divide the rock mass into strip-like block structures. In the study area, coal mining activities are conducted at the cliff toe of the steep ridge-type mountain geometry, resulting in the formation of extensive goaf areas. Combined with the steep and free-face topography of the mountain, a pronounced cantilever effect is generated. The tensile deformation of the overhanging rock mass further induces the development of tension cracks along the ridge crest. In addition, the pre-existing joints and faults cutting through the rock mass serve as intrinsic structural factors that facilitate tensile opening. Together, these factors jointly promote the formation of large-scale unstable rock masses, which constitute the initial driving mechanism of the dynamic “chain-reaction” process leading to surface cracking, toppling deformation, and even the progressive evolution into landslide–collapse hazards.

These tensile cracks exhibit two typical characteristics. First, the crack orientation is generally consistent with the ridge direction, and the cracks are wide at the surface but narrow downward, gradually tapering to extinction without directly connecting with the mining-induced fracture zone at depth. Second, deep and large tension cracks mainly develop along the ridge of the steep ridge-type mountain geometry, while their development toward the downslope areas on both sides gradually weakens.

**Figure 12:**
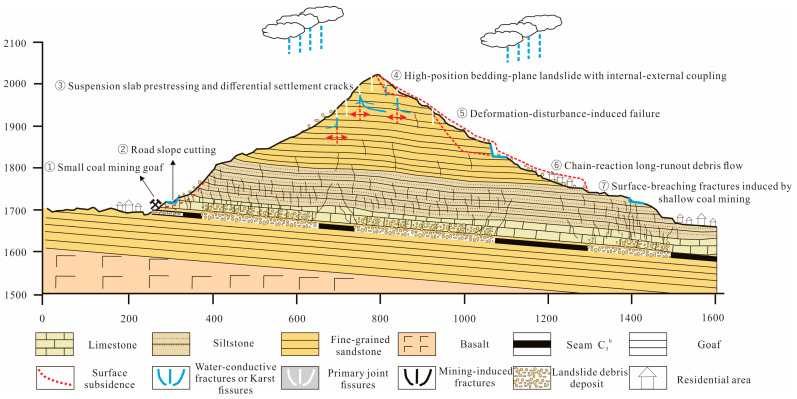
Conceptual model illustrating the mechanism of coal mining-induced geohazards in the steep ridge-type mountain geometry.

In the study area (Figure 12), the shallow overburden is directly penetrated by mining-induced deformation, which is prominently manifested by severe damage to the road surface. Coal seam extraction beneath steep slopes leads to the development of the typical “three zones” of mining-induced deformation, namely the collapse zone, the fracture zone, and the bending subsidence zone. In the shallow-buried zone within 50–150 m above the working face near the slope toe and gentle slope areas, the fracture zone and bending zone are directly connected with the jointed and weak sandstone–mudstone rock mass. This structural connection further triggers ground fissures, surface subsidence, and collapse–sliding failures.

Among these hazards, the mining-induced ground fissures directly cause severe road cracking, which is particularly evident on the hardened pavement in the study area. Moreover, the connection of surface fissures provides pathways linking the ground surface with underground mine workings, indirectly leading to abnormal ventilation airflow in underground return airways and establishing a direct hydraulic connection between surface rainfall infiltration and underground mine water inflow.

High-elevation deformation disturbances have developed in the study area, resulting in the formation of slope-unstable rock masses. At distances of approximately 150–400 m from the mining areas beneath the steep cliffs and steep slopes along the ridge crest, deformation within the mining-induced bending subsidence zone is transferred to this elevation. The slope gradients range from 40° to 60°, and the lithology is dominated by hard sandstone. Two sets of vertically oriented joints and unloading fractures are exceptionally well developed due to weathering and stress release, accompanied locally by natural rockfalls. Non-uniform deformation promotes the progressive propagation and interconnection of fractures, leading to two major failure modes: (i) blocky, columnar, and wedge-shaped rockfalls caused by rock mass segmentation, and (ii) the transformation of loosened surficial rock–soil materials into debris flows under rainfall erosion.

Disturbances induced by shallow historic mining activities at small coal adits and by road cut slopes have triggered small-scale historical ground fissures, landslides, and collapses near the coal seam outcrop on the western flank of Yueliang Mountain.

Long-term and large-scale underground mine drainage may induce a decline in the local groundwater table, which in turn triggers subsidence within shallow unconsolidated deposits. This effect is particularly pronounced in ancient (relict) landslide deposits at the slope toe, significantly increasing the damage risk to overlying residential buildings.

Ultimately, these processes give rise to a cascading secondary hazard chain, in which high-elevation rockfall and landslide deposits accumulate within gullies and, under catchment-scale runoff concentration, evolve into debris flows.

## 6. Conclusions

1. Even at burial depths of 200–300 m, coal mining in the mountainous regions of Southwest China can induce significant surface damage—including ground fissures, subsidence, collapses, and large-scale deformation—due to the amplification effects of steep slopes and specific lithological combinations.
2. Mining-induced damage does not necessarily evolve into large-scale slope failure of the mountain body. In most cases, the rock mass gradually regains stability through its own adjustment mechanisms. Fully understanding this phenomenon helps avoid overstating hazard risks in prevention and control practices and prevents excessive disaster mitigation measures.
3. Surface damage caused by coal mining can be accurately identified through field investigations, InSAR, and GNSS observations. These methods provide efficient and precise monitoring capabilities and should be widely promoted in mining hazard prevention.
4. Coal mining beneath a steep ridge-type mountain geometry exhibits a certain degree of lag between underground extraction and surface deformation. InSAR results show that the influence range is extensive, and severe subsidence zones are jointly produced by multiple working faces operating at different times.
5. A deformation–failure model specific to coal mining beneath steep ridge-type mountain geometry is identified. The unique morphology of the steep ridge-type mountain geometry and the underlying coal extraction exhibit strong coupling effects, making the region extremely susceptible to multiple geohazards. The combined impacts produce a complex disaster chain characterized by high-level collapses, slope failures, accumulation in gullies, and subsequent debris flows, revealing the complexity and high sensitivity of mining-induced geohazards in steep ridge-type mountain geometry.

## Figures and Tables

**Table 1:** The basic parameters of the SAR data used in this study.

Satellite	Band	Image Acquisition Date	Number of Scenes	Revisit Interval (Days)	Resolution (m), Azimuth × Range	Incidence Angle (°)
Sentinel-1	C	5 January 2022 to 1 January 2025	84	12/24	13.99 × 2.33	32.23

## Data Availability

The data used to support this study are available upon request from the author.
